# Estimate hidden dynamic profiles of siRNA effect on apoptosis

**DOI:** 10.1186/1471-2105-14-97

**Published:** 2013-03-15

**Authors:** Takanori Ueda, Daisuke Tominaga, Noriko Araki, Tomohiro Yoshikawa

**Affiliations:** 1 CytoPathfinder, Inc., 2-4-7 Aomi, Koto, Tokyo 135-0064, Japan; 2 Computational Biology Research Center (CBRC), National Institute of Advanced Industrial Science and Technology (AIST), 2-4-7 Aomi, Koto, Tokyo 135-0064, Japan

**Keywords:** siRNA, RNA interference, Prey–predator model, Ordinary differential equation, Parameter estimation, Genetic algorithm

## Abstract

**Background:**

For the representation of RNA interference (RNAi) dynamics, several mathematical models based on systems of ordinary differential equations (ODEs) have been proposed. These models consist of equations for each molecule that are involved in RNAi phenomena. Therefore, many real-value parameters must be optimized to identify the models. They also have many ‘hidden variables’, which cannot be observed directly through experimentation. Calculation of the values of the hidden variables is generally very difficult, if not impossible in some special cases. Identification of the ODE models is also quite difficult.

**Results:**

We show that the simplified logistic Lotka–Volterra model, a well-established ODE model for biological and biochemical phenomena, can represent RNAi dynamics as a predator–prey system. Although a hidden variable exists in the model, its values can be determined and made visible as dynamic profiles of RNA-decomposing effects of siRNAs. Correlation analysis shows that the model parameters correlate highly with the total effect of the siRNA.

**Conclusions:**

The results suggest that analyses using our model are useful to estimate dynamic profiles of siRNA effects on apoptosis and to score siRNA by its effects on apoptosis, namely ‘phenotypic scoring’.

## Background

In a body of living eukaryotic cells, RNA interference (RNAi) is a phenomenon caused by small interfering RNA (siRNA, a double-strand RNA consisting of 21–23 base pairs) in which there is decomposition of single-strand RNA that has a sequence that is complementary with the siRNA [1,2]. Artificially introducing siRNA into cells is widely used in experiments to suppress gene expression or to interrupt gene regulatory networks. Therefore, RNAi is a very useful and popular technique for approaching the molecular mechanisms of life.

Eukaryotic cells can incorporate external RNA. The incorporated double-strand RNA is then separated into two single strands that combine with protein molecules to form an RNA-induced silencing complex (RISC). The RISC combines with an RNA molecule in a cell that has a complementary sequence with the siRNA and decomposes it into fragments. When this target RNA is messenger RNA (mRNA), then either expression of the protein that is translated from the mRNA is suppressed or malfunctioning protein molecules will be produced.

The molecular mechanisms of RNAi are complex and are not completely revealed. In addition, quantitative measurements of the amount of incorporated siRNA and its effectiveness or strength of RNA decomposition are very difficult, especially in a time series. The authors believe that quantitative mathematical models can be applied to address this problem.

Several mathematical models have been proposed to represent the mechanisms and dynamics of RNAi [3-6]. These models are systems of linear ordinary differential equations (ODEs). Each equation in the system represents kinetics of a chemical reaction that constitutes the RNAi mechanism.

These ODE models to date are based on uncompleted (or partial) knowledge of RNAi mechanisms and consist of various quantities of parameters (Table 1). The model parameters are real numbers and can be determined by numerical optimization with sufficient computational power. However, this necessitates sufficient experimentally observed time series data for all variables in the model.

**Table 1:** Scales of previously proposed models

	**Equations**	**Parameters**
Fundamental model	4	12
Considering cell cycle	12	27
Considering viral effect	17	14
Self-targeting siRNA	4	8
Predator–prey model (**our model**)	2	4

Number of equations and parameters in mathematical models which have been proposed today. *Equations* denotes the number of differential equations used in each model. *Parameters* represents the total number of parameters in each model. The fundamental model represents the basic molecular mechanisms of RNAi [3]. The models considering the cell cycle [4], considering the viral effect [5], and with self-targeting siRNA [6] target the specific phenomena, as labeled. Our model is the abstract predator–prey model and is not based on the detailed molecular mechanisms.

From the perspective of numerical optimization, scales of these models, *i.e.* quantities of parameters, are not sufficiently small to identify models for actual amounts of available observed data. In addition, these models include hidden variables that are extremely difficult to observe. Hidden variables increase the degrees of freedom of the models, so quite large amounts of observation data are necessary to identify these models (to make the degrees of freedom of the model zero). Because experimental observation costs money, in many cases the time series data that are necessary to identify the models are insufficient.

The molecular mechanisms of RNAi are the main target of molecular biology today, and new knowledge about that has been growing. Previously established models did not consider such newly found mechanisms. Instead, abstract mathematical models need not change the model formulae.

A mathematical model for a system that has unknown mechanisms, such as for the RNAi phenomenon, can be expected to fit the observed data with fewer degrees of freedom (fewer number of parameters) in the sense of Ockham’s razor rather than those that fit better comparably but with more parameters.

RNAi is a phenomenon by which siRNA degrades target RNA molecules. This relation can be considered like that of prey and predator in a natural food chain even if the siRNA is artificially applied.

The Lotka–Volterra (LV) model is a very popular and widely applied ODE system for predator–prey systems. Many variations of the LV model have been proposed [7]. We introduce one of the simplest (fewest variables and parameters) of the modified models because the LV-based models can be identified without complete knowledge of molecular mechanisms of RNAi. When populations of prey and predator are represented as variables *x* and *y* respectively, the original LV model,

(1) $$\begin{array}{cc} \frac{\text{dx}}{\text{dt}} & =x(a-\text{by})\\ \frac{\text{dy}}{\text{dt}} & =y(\mathrm{cx}-d), \end{array}$$

does not consider the carrying capacity, which represents how many individuals can live in the given environment. Besides this, the LV model is unstable when the effect of the predator is very small. In such a case, the population of prey goes to infinity. These problems are solvable by introducing one term for each equation to make the system logistic form as follows:

(2) $$\begin{array}{cc} \frac{\text{dx}}{\text{dt}} & =x(a-\text{bx}-\text{ey})\\ \frac{\text{dy}}{\text{dt}} & =y(f-\text{cx}-\text{dy}). \end{array}$$

This model is called the logistic Lotka–Volterra (LLV) model [8].

Here we consider the apoptosis phenomenon that is triggered by the introduction of siRNA. When siRNA degrades its target, the cell dies by the apoptosis mechanism. The LLV model can represent this abstract scheme by assigning *x* to the number of living cells and *y* to the strength or killer effect of the siRNA. The number of cells is generally restricted by the carrying capacity of the environment because of physical conditions (decreased nutrition, accumulation of excrement, stacking of cells, etc.). Therefore, it should be represented by the logistic form. However, the strength of siRNA is not a physical variable and represents no actual or specific molecular mechanism. For that reason, it should be a dimensionless variable. We have no idea whether the logistic form (carrying capacity) should be applied. We then remove the logistic restriction from the equation for the predator in Equation 2 and apply it to the apoptosis by siRNA as

(3) $$\begin{array}{ll} \frac{\text{dx}}{\text{dt}} & =x(a-\text{bx}-\text{ey})\\ \frac{\text{dy}}{\text{dt}} & =y(\text{cx}-d), \end{array}$$

where *x* is the number of living cells, and *y* stands for the strength of siRNA that causes apoptosis (cell death). We will call this ODE system the Ueda model. The variable *y* is not observable, although *x* is observable using several experimental techniques. The hidden variable *y* is readily calculated using numerical integration when the observation data of *x* are given and the values of all five parameters (*a*, *b*, *c*, *d*, and *e* in Equation 3) and the initial value of the hidden variable (*y*_0_) are given. Therefore, the trial-and-error procedure, or heuristic optimization techniques, can find these parameter values by fitting *x* to the given time series data.

Here we show that the best-fitted models can clarify the dynamic profile of the invisible hidden variable (*y* in Equation 3) that implies the strength of the siRNA. We also show that its parameter values are distributed according to the strength of siRNA, and a parameter in the model that highly correlates with the total effect of siRNA. Model parameters are determined based on experimentally observed data. Our method and model proposed in this paper is for evaluation of siRNA, and is not for prediction. The model parameters quantitatively represent the strength of an siRNA, and can be interpreted as a kind of score value. This is the first approach to score siRNA by its effectiveness.

## Methods

### Negative control experiment

The study presented here models cell population changes that occur over time because of introduction of siRNA. Six commercial siRNA mixtures, each with a different strength of causing apoptosis, were modeled and compared. These siRNA molecules, which are commercial products developed to cause apoptosis in HeLa cells, are produced by Qiagen Inc., U.S.A., and are distributed by Dharmacon inc., U.S.A. (Table 2). The strength of siRNAs differs according to whether it is a mixture of multiple sequences, its sequences, its length, and the point of its targets in the apoptosis pathways. Therefore, the parameter values of the Ueda model differ for each of the siRNA product.

**Table 2:** Introduced siRNA to cause apoptosis on HeLa cells

**siRNA name**	**Product name**
ACD	QIAGEN:1027299 (AllStars Hs Cell Death Control siRNA)
KIF	QIAGEN:SI03019793 (Hs_KIF11_8)
PLK	QIAGEN:SI02223837 (Hs_PLK1_6)
VHP	QIAGEN:1027273 (Very High Potency Hs_CDC2 siRNA)
HP	QIAGEN:1027274 (High Potency Hs_CDC2 siRNA)
MP	QIAGEN:1027275 (Moderate Potency Hs_CDC2 siRNA)
Neg	QIAGEN:1027310 (Negative Control)

ACD is a cocktail of four siRNA sequences and has the strongest effect to cause apoptosis. The target of three siRNAs, VHP, HP, and MP, is common (the CDC2 gene), but they differ in strength. Neg is the non-targeting siRNA, which does not degrade any RNA in the cell.

**Figure 1:**
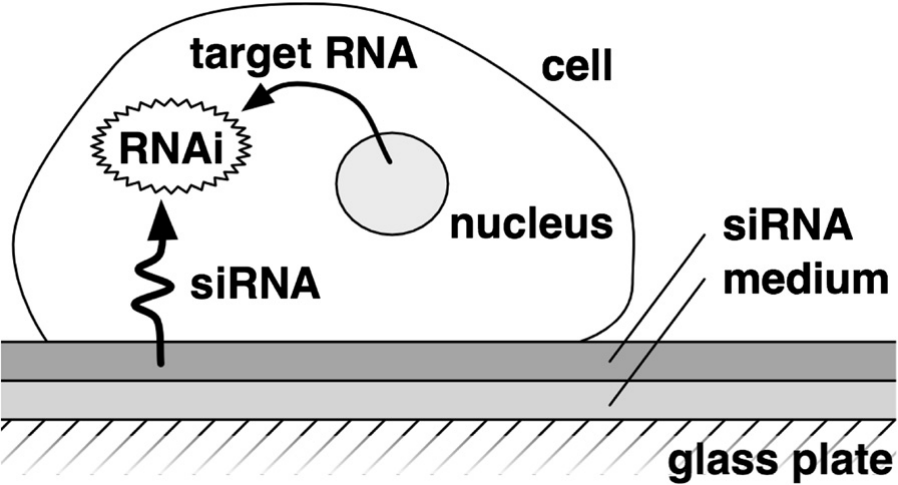
**Solid-state transfection technique.** The solid-state transfection technique that is used to introduce siRNA molecules into cells.

The solid-state transfection technique [9] was used to introduce siRNA into the cells (Figure 1). The ATP luminescence assay (a destructive measurement technique) [10] was used to measure the cell population. The CellTiter-Glo Luminescent Cell Viability Assay kit which is distributed by Promega Corp., U.S.A. was used for the ATP array.

First, we observed cell population changes with the negative control (Neg) siRNA. No RNA molecule was degraded by introducing the Neg siRNA; therefore, the strength of the siRNA in the Ueda model (Equation 3), *y*, was kept at zero. In this case, the Ueda model consists of only one equation with parameters *a* and *b*, as

(4) $$\frac{\text{dx}}{\text{dt}}=x(a-\text{bx}).$$

The times of observations were at 2.4, 5.4, 8.0, 20.7, 29.0, 44.9, 52.7, 68.7, 76.8, and 92.7 hr after the start of cultivation (ten time points in total). The siRNA introduction started simultaneously with cultivation. The measured values were the intensity of fluorescence in the ATP assay, not the actual numbers of cells. The mean value of four repeated experiments was used as the data for each point of the sampling time.

The parameter estimation in this negative control experiment was optimization of three real parameters (*a*, *b*, and *x*_0_) from ten real-number data points at the designated times. The real coded genetic algorithm was used to find the optimal values of the parameters (explained below).

**Figure 2:**
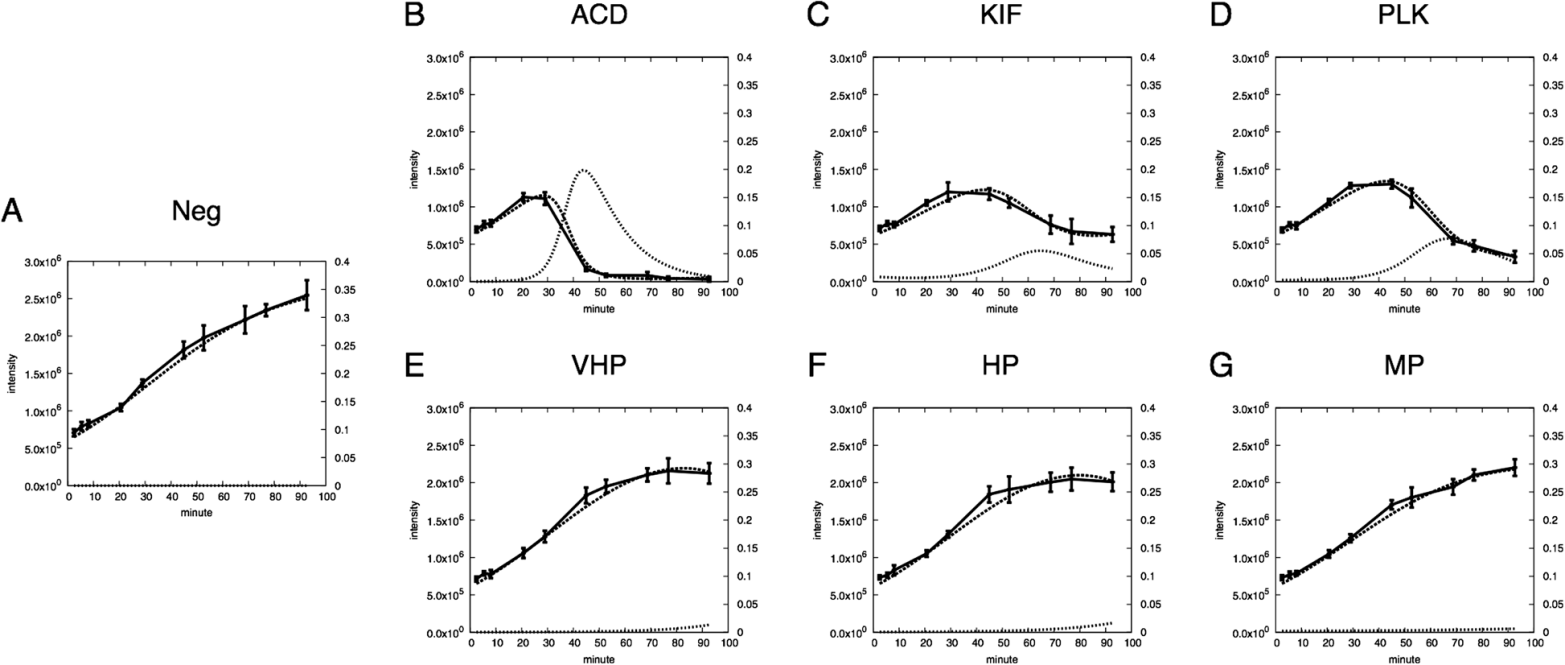
**Time courses of cell population in apoptosis.** Cell population change in time under the conditions of negative and positive controls (**A**–**G**, solid) and curves of the optimized Ueda model for each condition (**B**–**G**, dash – cell population; dot – siRNA effect). Neg siRNA does not cause RNAi in the negative control condition. Therefore no dotted line is shown in A.

The observed cell population and the fitted curve are shown in Figure 2A. The observed profile in the negative control condition shows the typical growth curve (logistic curve) of microbial cultivation. Values of the parameters *a* and *b*, and *x*_0_, the estimated initial value of *x*, are shown in Table 3.

**Table 3:** Estimated model parameters

**siRNA**	***a***	***b***	***x***_**0**_	***c***	***d***	***y***_**0**_
Neg				-	-	-
ACD				2.694e-7	8.087e-2	2.947e-4
KIF				1.784e-7	1.506e-1	8.382e-3
PLK	3.949e-2	1.445e-8	6.512e+5	1.337e-7	9.045e-2	2.837e-3
VHP				2.498e-8	3.091e-7	3.458e-4
HP				2.243e-8	1.118e-7	6.760e-4
MP				7.258e-9	6.509e-8	2.305e-3

Estimated parameter values of the Ueda model for each siRNA condition. Values for *a*, *b*, and ***x***_***0***_ are common for all conditions. Values of *c*, *d*, and ***y***_***0***_ are undefined for the negative control condition.

### Positive control experiment

Next, we introduced siRNA into HeLa cells to cause apoptosis and observed the changes of the cell population in time. Experimental conditions were the same as those of the negative control observation except that effective siRNA was induced into the cells. The observed cell population changes and fitted curves are shown in Figure 2B through 2G.

The values of the parameters *a* and *b* and *x*_0_ are the same as those estimated at the negative control experiment. Therefore, the number of estimated values for the model of the positive control experiments is three (*c*, *d*, and *y*_0_), and the numerical difficulties of this optimization are the same as in the case of the negative control experiment. The values of the estimated parameters, *c*, *d*, and *y*_0_, are shown in Table 3 for each siRNA.

### Numerical optimization

Parameter optimization of this case study was accomplished by finding the parameter values that made the curve of the model best fit the given data. The fitness of each model was calculated as the reciprocal of the total sum of the squared relative error between the given data and the model curve, which was calculated using numerical integration. We used the Runge–Kutta method (4th order) as the method of numerical integration.

The optimization task was undertaken to maximize the fitness of the model. We used the real coded genetic algorithm [11], which introduces UNDX [12] as the crossover operation and MGG [13] as the selection operation. The estimated optimal parameter values are shown in Table 3.

## Results and discussion

### Estimation of models

The effects of the siRNAs are not defined exactly in common, however, and they are considered ideally as reflecting the rate of the degradation of the target RNA molecules. Assuming that it is reflected in the cell population in our case study, the area under the siRNA strength curve, which is calculated using numerical integration, can be interpreted as the actual total of the siRNA effect. The differences between the curves for cell populations with and without siRNA (positive and negative controls) are also regarded as the siRNA effect.

We then compared the estimated model parameter values with the accumulated differences between the curves of the cell populations with and without siRNA, the accumulated siRNA curves, and the highest points of the siRNA strength curves (Table 4). Principal component analysis (PCA) conducted on the parameter values of *c* and *d* shows that the parameter *d* is most likely to be significant in distinguishing the siRNAs (the loadings of the parameter values on the first principal component, PC1 in Table 4, highly correlate with parameter *d*). However, Pearson’s correlation coefficients indicate that parameter *c* is highly correlated with the total effect of the siRNAs 0.976 for accumulated differences between with and without siRNA, and 0.968 for siRNA strength.

**Table 4:** Correlation between measurements calculated from the estimated dynamics of siRNA strength

	**Diff.**	**siRNA**	**Height**	***c***	***d***	**PC1**
Diff.	1.0	0.99922	0.97268	0.97605	0.68269	0.69267
siRNA		1.0	0.97307	0.96764	0.66965	0.67984
height			1.0	0.92129	0.50365	0.51537
*c*				1.0	0.79264	0.80059
*d*					1.0	0.99989
PC1						1.0

Peason’s correlation coefficients between the area under the curve (AUC) for differences in the cell population between siRNA treatments and the negative control (Diff.), AUC of siRNA strength (siRNA), the highest points of siRNA strength curves (height), model parameters *c* and *d*, and the loading values of siRNAs on the first principal component (PC1), as derived from six values of each *c* and *d* by PCA.

**Figure 3:**
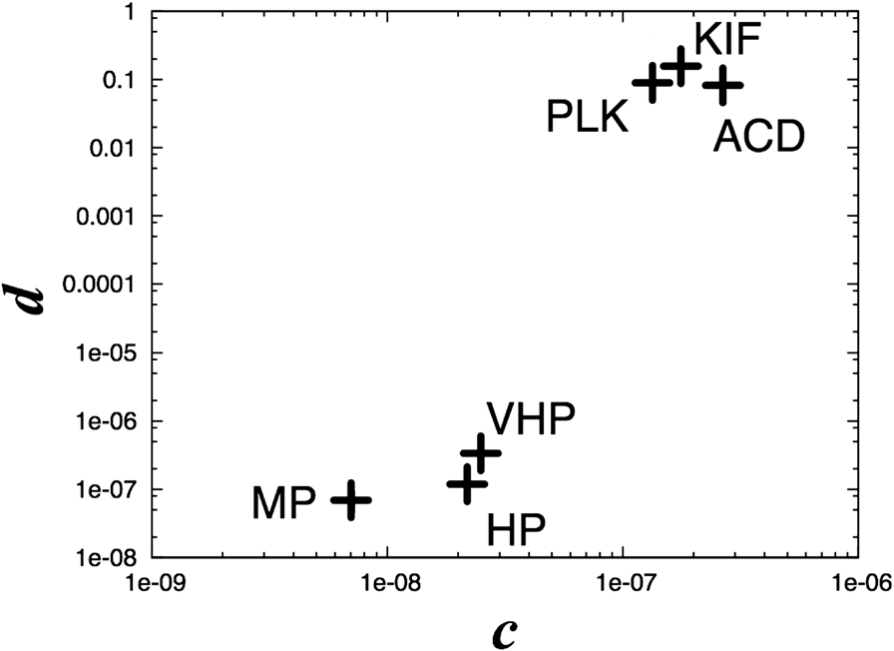
**Estimated model parameters.** Estimated values of parameters *c* and *d* in the Ueda model for siRNA, which causes apoptosis in HeLa cells.

Scatter plots of the parameters *c* and *d* of the Ueda model for siRNAs that induce apoptosis are shown in Figure 3. The shown parameters of siRNAs are apparently classified into two groups, namely the strong-effect group (ACD, KIF, and PLK) and the weak group (VHP, HP, and MP). This figure clearly illustrates that estimation of fitting the Ueda model can be used to classify siRNA by its actual target decomposition strength.

### Discussion

The parameter estimation problem of the Ueda model in this case study is similar to the least squares method that minimizes the total sum of absolute errors, whereas our optimization minimizes relative errors. The amount of data (10 sampling points) is considered sufficient to estimate the number of parameters (2 parameters and 1 initial value, total is 3).

The profile of the cost function to be minimized (total sum of the relative error between the given data and the time series data calculated using numerical integration of the model) has a highly nonlinear shape because the model cannot be solved numerically in certain regions of the parameter space. In such regions, numerical computational errors such as overflow, underflow, and division by zero prevent calculation of the cost function. Established analytical optimization methods such as the steepest descent method, the conjugate gradient method, arrangements of the Newton–Raphson method, etc. require smooth search regions that do not contain such regions. However, determining those regions before optimization is extremely difficult in most cases. Reportedly, heuristic search methods are effective for optimization of systems of differential equations [14].

Correlation analysis shows that the parameter *c* is highly correlated with total siRNA effects. This fact suggests that the value of *c* can be used to score the siRNAs. Larger values of *c* signify a stronger effect. This score reflects the actual effect without considering the nucleic acid sequence of siRNA that relates its characters. Therefore, this score can be called a ‘phenotypic score’. Further analysis of additional experimental data is needed to prove the reliability of this score.

According to the general interpretation of the LLV model, the parameter *b* in the Ueda model is understood as the carrying capacity of the environment in which the cells are living. Here, *c* represents the positive effect of the cell population to the strength of the siRNA. Because intake of external siRNA is more active on more viable cells, *c* might be interpreted as the siRNA-inducing capacity of the cells, or more generally, the cell viability under the cultivating condition.

Each term of the Ueda model can be interpreted as follows: *ax* represents self-reproduction rate of cells, −*bx* denotes the carrying capacity, −*exy* is degradation rate of cells by Apoptosis caused by siRNA, *cxy* is siRNA incorporating rate to cell bodies that reflects cell activity or viability, and −*dy* is the decreasing rate of siRNA effect by decreasing cell viability.

One difficulty of the Ueda model currently is the fitting difficulty. Results demonstrated that multi-point heuristic searches are effective for systems of ODEs [11,14]. However, this optimization method demands many computational resources. To apply a fast analytic search, the cost function should be modified.

Long simulation of the Ueda model also should be considered. After the end point of the observation the model shows an oscillating profile. Continuous experiments could not match the oscillating profile of the simulation because siRNA molecules in the medium would be exhausted.

The Ueda model is not based on molecular mechanisms. The S-system [15] is the same on this point. Applying this canonical form ODE model to RNAi dynamics is expected to be interesting for its capability for application to dynamical system analyses.

## Conclusions

Cell population changes by apoptosis that results from introduction of siRNA were observed as quantitative time course datasets. The Ueda model, the simplified logistic Lotka–Volterra model, can fit these datasets. The optimal models represent dynamic profiles of RNA decomposing effects of siRNA in apoptosis that cannot be observed directly through experimentation. Parameter estimation using the Ueda model can be done using the real coded genetic algorithm. One estimated parameter correlates highly with the estimated siRNA strength. We think that this parameter might represent the effectiveness of siRNA.

## Abbreviations

LV model: Lotka–Volterra model; LLV model: Logistic Lotka–Volterra model; mRNA: messenger RNA; ODE: Ordinary Differential Equation; PCA: Principal Component Analysis; RISC: RNA-Induced Silencing Complex; RNAi: RNA interference; siRNA: short interfering RNA.

## Competing Interests

The authors declare that they have no competing interests.

## Authors’ contributions

TU devised the mathematical model, made parameter optimization software, and carried out the optimization of the model. DT analyzed optimized models, considered results and wrote the manuscript. NA and TY planed and carried out experimental observations. All authors read and approved the final manuscript.
